# Outcome of proximal esophageal cancer after definitive combined chemo-radiation: a Swiss multicenter retrospective study

**DOI:** 10.1186/s13014-017-0834-8

**Published:** 2017-06-14

**Authors:** Evelyn Herrmann, Nando Mertineit, Berardino De Bari, Laura Hoeng, Francesca Caparotti, Dominic Leiser, Raphael Jumeau, Nikola Cihoric, Alexandra D. Jensen, Daniel M. Aebersold, Mahmut Ozsahin

**Affiliations:** 1 Department of Radiation Oncology, Bern University Hospital, and University of Bern, Freiburgstrasse, CH-3010 Bern, Switzerland; 2 Department of Radiation Oncology, Lausanne University Hospital, Lausanne, Switzerland; 3 Department of Radiation Oncology, Kantonsspital St. Gallen, St. Gallen, Switzerland; 4 Department of Radiation Oncology, Geneva University Hospital, Geneva, Switzerland; 5 Department of Diagnostic, Interventional and Pediatric Radiology, Bern University Hospital, University of Bern, Bern, Switzerland; 6 Radiation Oncology Department, Besançon University Hospital, Besançon, France

**Keywords:** Proximal esophageal cancer, Esophagus, Radiotherapy, Chemotherapy

## Abstract

**Objective:**

To report oncological outcomes and toxicity rates, of definitive platin-based chemoradiadiationtherapy (CRT) in the management of proximal esophageal cancer.

**Methods:**

We retrospectively reviewed the medical records of patients with cT1-4 cN0-3 cM0 cervical esophageal cancer (CEC) (defined as tumors located below the inferior border of the cricoid cartilage, down to 22 cm from the incisors) treated between 2004 and 2013 with platin–based definitive CRT in four Swiss institutions. Acute and chronic toxicities were retrospectively scored using the National Cancer Institute’s Common Terminology Criteria for Adverse Events, version 4.0 (CTCAE-NCI v.4.0). Primary endpoint was loco-regional control (LRC). We also evaluated overall survival (OS) and disease-free survival (DFS) rates. The influence of patient- and treatment related features have been calculated using the Log-rank test and multivariate Cox proportional hazards model.

**Results:**

We enrolled a total of 55 patients. Median time interval from diagnosis to CRT was 78 days (6–178 days). Median radiation dose was 56Gy (28–72Gy). Induction chemotherapy (ICHT) was delivered in 58% of patients. With a median follow up of 34 months (6–110months), actuarial 3-year LRC, DFS and OS were 52% (95% CI: 37–67%), 35% (95% CI: 22–50%) and 52% (95% CI: 37–67%), respectively. Acute toxicities (dysphagia, pain, skin-toxicity) ranged from grade 0 – 4 without significant dose-dependent differences. On univariable analyses, the only significant prognostic factor for LRC was the time interval > 78 days from diagnosis to CRT. On multivariable analysis, total radiation dose >56Gy (*p* <0.006) and ICHT (*p* < 0.004) were statistically significant positive predictive factors influencing DFS and OS.

**Conclusion:**

Definitive CRT is a reliable therapeutic option for proximal esophageal cancer, with acceptable treatment related toxicities. Higher doses and ICHT may improve OS and DFS and. These findings need to be confirmed in further prospective studies.

## Introduction

Cervical esophageal cancer (CEC), located between the cricopharyngeal muscle and the sternal notch, represents < 5% of all esophageal cancers [1]. In contrast to the middle and lower esophageal cancer, for which chemo-radiotherapy (CRT) is the standard of care, for both, in the neo-adjuvant and definitive setting [2–5], the management of CEC remains controversial. In the past, patients with CEC underwent surgery, including pharyngo-laryngo-esophagectomy [6], resulting in permanent tracheostomy, impacting the quality of life of these patients enormously [7]. Five-year overall survival (OS) rates with surgery alone are poor, ranging between 12 – 27%, while operative morbidity is substantial (29–55%) [1]. Due to its anatomical proximity to the hypopharynx, as well as its common etiology, CEC is usually treated in analogy to hypopharyngeal cancer [8] or standard esophageal cancer protocols [9]. Randomized trials of esophageal cancer and squamous-cell carcinoma (SCC) of the head and neck have demonstrated improved survival with CRT compared to radiotherapy (RT) alone. However, these trials have not included CEC [6, 9–11]. There are no prospective clinical trials to guide treatment in CEC. Retrospective studies evaluating the role of definitive CRT are scarce [6, 12–19]. Five-year OS following definitive RT alone ranges between 15 and 32% [1], while definitive CRT can achieve 5-year OS rates up to 55% with mean total radiation doses ranging from 61.2 to 66Gy, with acceptable toxicity [20]. As a result, RT or CRT have emerged as the preferred treatment modalities for SCC of the upper esophagus. However, no consensus has been reached, with regards to the optimal sequence of CRT, nor which RT dose should be delivered. A lot of patients are treated using institutional protocols. The aim of the current multicenter study is to report the oncological outcome and toxicity rates of definitive external beam RT combined with platin-based chemotherapy, with a particular focus on the impact of RT dose and sequence of chemotherapy (CHT).

## Materials and methods

Medical records of patients with CEC treated between 2004 and 2013 with definitive CRT in four Swiss institutions were retrospectively analyzed. The analysis included non-metastatic patients with a pathologically confirmed CEC. We defined a CEC as a tumor of the esophagus located between the inferior border of the cricoid cartilage to 22 cm from the incisors. Patients with prior CRT or secondary cancers either synchronously or within the past five years were excluded. All patients were treated according to institutional protocols, consisting of either platin-based induction chemotherapy (ICHT), concurrent platin-based CRT or both. If ICHT was administered, a platinum-based regimen [21] was used. Concurrent chemotherapy was administered using regimens that included cisplatin and 5-fluorouracil (5-FU), oxaliplatin and 5-FU or carboplatin and paclitaxel [22, 23]. If the Cisplatin/5-FU regimen was given, each cycle of CHT was given on days 1 and 29 and 5-FU was administered as a continuous intravenous infusion after completion of the cisplatin on days 1 through 4 and 29 through 32. Concurrent platin plus taxane based CHT was given weekly up to 6 cycles. Target delineation was based on international consensus guidelines [24]. Contouring was carried out on CT scans with a slice thickness of 2–3 mm and included information from PET scans and endosonography.

### Toxicity assessment and follow-up

Patients were clinically assessed on a weekly basis during CRT, at which time point laboratory parameters (such as hemoglobin, leukocytes, platelets and renal function) were reviewed. The first clinical follow up visit was performed 4 to 6 weeks after completion of treatment, afterwards, every three months in the first two years, six months the third year and annually thereafter. Each follow-up included a physical examination and blood work (hemoglobin, leukocytes, platelets, renal function and hepatic function). Diagnostic CT including neck/thorax/abdomen and endoscopy took place every six months during five years and thereafter annually. PET/CT was performed, when clinically indicated. All observed adverse events were graded according to the National Cancer Institute’s Common Terminology Criteria for Adverse Events, version 4.0 (CTCAE-NCI v.4.0) [25].

### Statistical analysis

Pseudonymized multi-institutional patient data were pooled in a central database. Time to event data was calculated from the first day of RT until the last follow up or until death using the Kaplan-Meier method. Loco-regional control (LRC) was defined as the absence of tumor progression in the treatment volume on follow-up. Disease-free survival (DFS) was defined as time until local or distant disease relapse after treatment or death due to any cause. Overall survival was defined as time from diagnosis until death from any cause The Log-rank test was used for univariable analysis (UVA) for continuous prognostic factors, the median value was used for grouping and the Cox proportional hazard model was used for multivariable analysis (MVA). The Linear-by-Linear Association test was used to compare toxicities. A significance level of p = 0.05 was used; all tests were two- sided. Factors having a p-value ≤ 0.20 in UVA and technical related factors of interest, such as RT dose, have been included in MVA. The MVA models for LRC, DFS and OS were created using backward selection. Statistical analysis was performed using JMP 10.0 statistical software (Cary, NC).

### Ethics

All patients gave informed consent prior to initiation of treatment. Research ethics board approval was obtained for this analysis (PB_2016-01147). This work is in accordance with the Declaration of Helsinki in its most recent version.

## Results

### Patients’ characteristics

Fifty-five patients were meeting the inclusion criteria of this study. Forty-two patients (76%) were male and the mean age was 64 years (42–78 years). Median follow-up was 34 months (6-110 months). Baseline patient characteristics are shown in Table 1. During the investigated period (2004-2013) the irradiation technique changed how esophageal carcinomas are treated. It shifted from 3-D to IMRT technique. Fig. 2 gives an overview of the patients treated per year. Yet, some patients continued to be treated with 3-D technique. Ninety percent of patients presented with dysphagia before treatment, scored as grade 1, 2 and 3 in 36%, 40% and 14%, respectively. The majority of patients presented with a locally advanced tumor (76% cT3-4 and 67% cN+). Median primary tumor length was 5 cm (1 – 14 cm). All but four patients had biopsy-proven SCC; the remaining patients presented a carcinoma in situ (*n* = 1), an adenocarcinoma (*n* = 1), and in two patients histology was not conclusive.

**Table 1:** Baseline Characteristics

Characteristic	Number of patients [N]
Age (years), median (range)	64 (42-79)
Gender	
Male	42
Female	13
Pathological grade	
G 1-2	21
G 3	11
Gx/NA	23
Pathology	
Squamous cell	52
Adenoid cell	1
CIS/NA	1/1
T stage	
≤ 2	12
3-4	42
Tx	1
N stage	
N 0	18
N 1-3	37
TNM stage	
I-II	20
III	34
NA	1
Radiation dose (Gy)	
< 56	26
≥ 56	29
Radiotherapy technique	
3D	14
IMRT	24
Tomotherapy	17
Induction chemotherapiy	
Yes	32
No	23
Concurrent chemotherapy	
Yes	51
No	4
Acute Tox ≥ Grade 3	
Dysphagia	8
Skin	3
Pain	7
Haematological	5
Chronic Dysphagia	
Grade 1-2	30
Grade 3-4	5
NA	20
Patients by center	
Bern University Hospital	16
Hôpitaux Universitaires de Genève	7
Centre Hospitalier Universitaire Vaudois	16
Kantonsspital St.Gallen	16

### Radiotherapy

Median time interval from diagnosis to RT was 78 days (6-178 days). RT was delivered either in a single, or two courses (boost). Single doses from 1.2 to 5Gy were used. Twenty-one patients (38%) were irradiated using conventional 3-D, and 34 patients (62%) with intensity-modulated RT (IMRT). Median cumulative RT dose was 56Gy (28–72Gy). Fifty-three patients (96%) received external beam RT alone. Two patients (4%) received 50.4Gy, using IMRT with a boost delivered using high dose rate brachytherapy (HDR-BT) (2 × 3Gy), up to a total dose of 56.4Gy.

### Chemotherapy

Thirty-two patients (58%) received induction chemotherapy (ICHT), mainly delivered with platin-based regimens (cisplatin, *n* = 10, carboplatin *n* = 22) combined with 5-fluorouracil (5-FU, *n* = 10) or with taxanes (*n* = 22). Median number of cycles ICHT was two (0–4 cycles). The same CHT regimens were delivered concurrently to RT in 51 patients (93%). Four patients did not receive concurrent CRT because of hematological toxicities after ICHT (*n* = 3) and patient refusal (*n* = 1).

### Toxicity

Most frequent radiation-related acute toxicities included dysphagia, pain and skin-reactions. Grade 2 dysphagia occurred in 45% of patients. Higher-grade dysphagia (grade ≥3) was reported in 15% of patients. Eighty-two percent of patients experienced pain (odynophagia) throughout radiotherapy. Grade 1, 2 and 3 odynophagia were reported in 42 , 27 and 12%. ICHT had no impact on odynophagia (*p* = 0.76). Unfortunately, no data on pre-treatment odynophagia levels were available. Acute skin toxicity was assessed in all patients, grade 1, 2 and 3 skin reactions were reported in 29 , 15 and 5%, respectively. The remaining patients (51%) showed no signs of acute skin toxicity. In the group of patients, which received ICHT, only 6% presented with a grade 2 and 3% with a grade 3 skin reaction, whereas 26% of patients treated without ICHT had a grade 2 and 9% had a grade 3 skin toxicity, even though no statistical difference was found in the two groups (*p* = 0.05).

Grade 1, 2 and 3 CHT-associated hematological toxicities were reported in 20 , 33 and 9% of the population, respectively. Within the group of patients with ICHT, grade 2 hematological toxicities were significantly higher (*p* = 0.04), but no differences in incidence of severe (grade 3+) toxicity were seen (11 vs. 13%, *p* = 0.87).

Only two patients (4%) needed hospitalization for treatment-related toxicity (one patient for uncontrolled dysphagia and one for tumor bleeding). One patient (2%) was hospitalized for his brachytherapy boost and one patient (2%) was hospitalized because of installation of a Witzel fistula.

At last follow up, 33% of patients had no signs of dysphagia; grade 1, 2 or 3 dysphagia was observed in 13%, 11% and 9%. Noteworthy, no data on late esophageal toxicity were available in 18 patients (32%).

### Patterns of failure

Thirty-three patients (60%) had developed a treatment failure consisting of 31% isolated local failure (*n* = 17), 11% isolated systemic failure (*n* = 6) and 29% combined local and distant failure (*n* = 16), Noteworthy, 38% (*n* = 21) of patients had no treatment failure. In one patient (2%) no data were available about patterns of failure.

### Treatment outcome

**Fig. 1:**
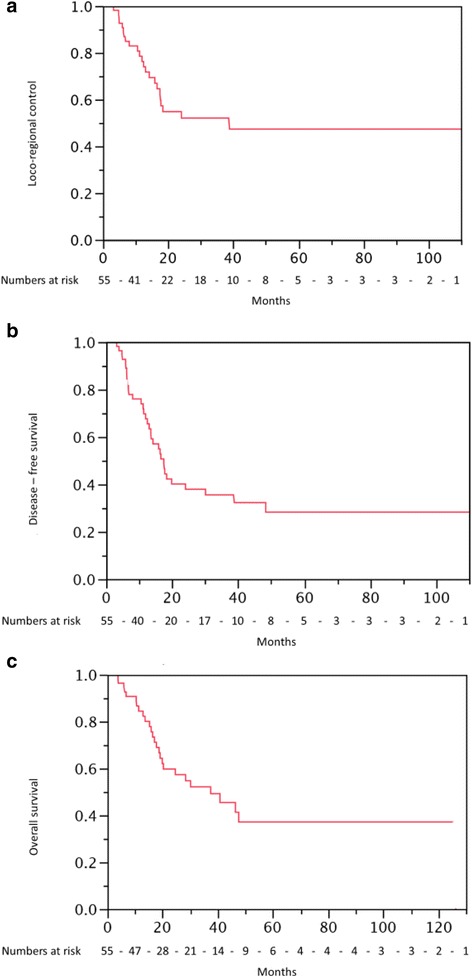
**a**-**c** Kaplan-Meier curves for **a**) Loco-regional control (LRC) **b**) overall survival (OS) C) disease-free survival (DFS)

**Fig. 2:**
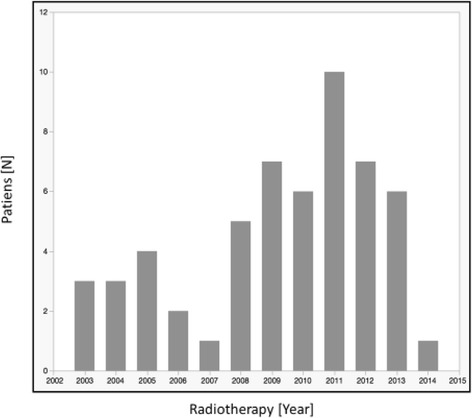
Treated patients per year

At last follow up, 28 patients (51%) were still alive. Three-year actuarial LRC, DFS and OS in the total cohort were 52% (95% CI: 37–67%), 35% (95% CI: 22–50%) and were 52% (95% CI: 37–67%), respectively (Figs. 1, 2 Kaplan-Meier curves for LRC, OS, DFS) For the patients receiving ICHT (*n* = 32), three-year LRC, DFS and OS were 60 , 43 and 60%, respectively. Three-year LRC, DFS and OS for patients without ICHT (*n* = 23) was 40 , 25 , and 40%, respectively. The difference in outcomes was not statistically significant (*p* > 0.10), since the number of patients in each group (ICHT vs. non-ICHT group) was small. On UVA (Table 2) longer time interval (>78 vs. <78 days) from diagnosis to CRT was a significant predictive prognostic factor for DFS (*p* =0.03). After backwards selection, MVA (Table 3) revealed that cumulative radiation dose >56Gy and ICHT were independent positive predictive factors for DFS [(*p* < 0.03) and (*p* < 0.02), respectively], and OS [(*p* < 0.006) and (*p* < 0.004), respectively]. T and N categories were not statistically significant predictive prognostic factors for LRC, DFS or OS (*p* >0.05).

**Table 2:** Univariate analysis of prognostic factors influencing OS, DFS, and LRC in cervical esophageal cancer

Factor	3-year LRC (%)	*p*-value	3-year DFS (%)	*p*-value	3-year OS (%)	*p*-value
Gender						
Female	51	0.71	17	0.28	42	0.2
Male	53		42		56	
Age						
≤ 65y	66	0.25	41	0.82	54	0.64
≥ 65y	39		30		52	
Tumor grade						
G2	55	0.14	44	0.34	68	0.12
G3	57		32		46	
TNM						
T1-2	54	0.62	26	0.27	56	0.7
T3-4	45	0.50	39	0.42	40	0.81
N0	57		44		49	
N+	50		31		53	
ICHT						
No	41	0.18	25	0.11	40	0.11
Yes	60		43		60	
Time to RT						
≤ 78days	43	0.24	24	0.03	53	0.45
≥ 78days	59		46		52	
RT Modality						
3-D	47	0.5	36	0.68	60	0.75
IMRT	55		36		47	
Dysphagia before treatment						
0	53	0.67	53	0.83	53	0.51
G1	57		34		48	
G2	57		36		59	
G3	29		25		37	
RT dose						
≥ 56Gy	56	0.76	36	0.78	56	0.88
≤ 56Gy	50		35		49	

*ICHT* induction chemotherapy, *RT* radiation treatment, *LRC* loco-regional control, *DFS* disease-free survival, *OS* overall survival

**Table 3:** Multivariate analysis of prognostic factors related to DFS, and OS in cervical esophageal cancer

Endpoint	Variable	HR	95% CI for HR	*p*- value
DFS	IDCHT	0.42	0.20 - 0.88	0.02
	RT Dose	0.95	0.9 – 0.99	0.03
OS	IDCHT	0.26	0.1 - 0.65	0.004
	RT Dose	0.01	0.0006 - 0.3	0.006
	Grade	0.02	0.002 - 0.46	0.033

*OS* overall survival, *DFS* disease-free survival, *IDCHT* induction chemotherapy, *RT Dose* radiation treatment dose, *95% CI* 95% confidence interval, *HR* hazard ratio

## Discussion

In the present study, we report results from a multicenter cohort of CEC patients treated with definitive CRT, with or without ICHT. No prospective clinical trials exist in CEC to guide treatment. Few retrospective studies were published, illustrating the outcomes of definitive CRT in CEC. These series were heterogeneous in terms of RT techniques, CHT regimens, and radiation doses. Approximately 59% of patients within these reports [6, 13, 15, 19, 26–28] received RT alone, and 41% received CRT [7, 13, 14, 16, 19, 26–29]. When a concurrent treatment approach was chosen, 22% of these patients received ICHT [16, 20, 29]. Three-year OS rates of 24–58% were reported in CEC patients treated with definitive RT, with or without CHT following short-term observation [12, 15, 16, 20, 29]. Table 4 gives an overview of the so far published outcome data of CEC patients treated with RT alone or RCT with or without ICHT. In our cohort, 3-years actuarial LRC, OS and DFS were 52% (95% CI: 37–67%), 35% (95% CI: 22–50%) and 52% (95% CI: 37–67%), respectively (Fig. 1a–c), and consequently is well comparable with the existing data. Moreover, longer time interval (≥78 vs. <78 days) from diagnosis to RT was a significant prognostic factor for DFS (*p* = 0.03). Since 58% of patients (*n* = 32) in our study have received ICHT, which prolongs time interval to CRT, we hypothesize ICHT could be an indicator for its impact on DFS. Therefore, we have included ICHT into MVA. After backwards elimination, ICHT was a significant predictive factor for DFS (*p* < 0.02) and OS (*p* < 0.004).

**Table 4:** So far published literature on RT +/- CHT in proximal esophageal cancer

Author (publication year)	Number of patients [N]	Definition of CE	Type of treatment (RT vs RCT)	IDCT (NO vs type of ICHT)	Dose of RT (total dose/fraction) [Gy/Gy]	LC [%/time]	Acute Toxicity ≥ G3 [N]	Late Toxicity ≥ G3 [N]	Prognostic factors	Surgery [N]
Mendenhall et al; 1988 [1]	34	NA	RT	NO	47-75/1.7-1.9	25.80%	4	4	Gender	3/34 Salvage surgery
Stuschke et al; 1999 [16]	17	between cricoid and upper thoracic inlet	RCT	Leucovorin + 5FU + Cis ± Etoposide	50/2; Boost 10/2 or 15/2x1.5; 2. Boost HDR 2x4	33%/2years; 19%/3years	4	0	NA	NO
Burmeister et al; 2000 [12]	34	between cricoid and upper thoracic inlet. Extension allowed	RCT	NO	50.4-65/NA	88%	12	5	NA	NO
Yamada et al; 2006 [15]	27	NA	RCT (23)	NO	44-73.7/1.8-2	52%	15	0	Performance Status/Tumor lenght	NO
Wang et al; 2006 [18]	35	tumor located above the carina	RCT	Various: platinum-based + 5-FU/paclitaxel/etoposide	24.5-64.8/1.8	47.7%/5years	NA	NA	Dose (>50Gy)	NO
Uno et al; 2007 [14]	21	cricopharyngeal muscle to thoracic inlet	RCT	NO	40/2; Boost 20-34/NA	52%	9	NA	T-Stage, initial LC	5/21
Huang et al; 2008 [13]	71 (50 curative intent)	NA	RCT	NO	54/2.7 or 50-56/2 + Boost 14-20/2	37%/47% (curative group)/2years	24/71	NA	Gender, Age (>64)	NO
Chou et al; 2010 [42]	29 (14 RT)	NA	RCT	NO	65 (60-75)/1.8-2	NA	NA	NA	NA	15/29
Ma et al; 2011 [43]	33 (69 upper thoracic esophagus)	NA	RCT	NO	50.4 + Boost 9 or 59.4/each 1.8 (41.4), then 2x1.5 (18)	80%/86%/3years	61	28	NA	
Tong et al; 2011 [7]	107 (21 RT)	NA	RCT	NO	40-46 or 60-68/2	NA	8	NA	NA	68/107
Cao et al; 2015 [28]	115	NA	RT (80)/RCT (35)	NO	59.4-76/1.8-2.12	83%/2years	28	2	Dose (>66 Gy)	10/115
Gikka et al; 2013, [20]	55	cricopharyngeus muscle to thoracic inlet (ca. 15 - 18 cm from the incisors)	RCT	5FU + leucovorin + Cis + etoposide; 5FU + leucovorin + Cis; Cis + irinotecan or taxanes	49.8-50.4/1.8-2; Boost 56-70Gy/1.5 2×/d; HDR 2× 4-5, or 1× 7	55%/2years; 47%/5years	59	11	NONE	NO
Tu et al; 2013 [44]	36	esophagus above tracheal eminence, and 24 cm from incisor teeth	RCT	NO	52-70/1.8-2	NA	28	NA	NA	NO
Cao et al; 2014 [26]	224 (133 RT/28 preOP-RT/postOP-RT 36)	NA	RT/RCT	NO	RT 6-80; RCT 28.8-76; preOP 40-50, postOP 45-60/1.8-2.12;	69.9%/2years	50	NA	Stage	63/224
Ludmir et al; 2014 [45]	37	between the upper esophageal sphincter and the thoracic inlet	RCT	NO	14.4-71/NA	65.6%/5years	NA	NA	NA	NO
Cao et al; 2015 [27]	101	NA	RT/RCT	NO	60-80/1.8-2.12	67.4%/2years	5	3	Age, Hoarseness	NO
Zhang et al; 2015 [29]	102	cricopharyngeal muscle to thoracic inlet	RCT	platinum-based (18)	50-70/NA	35.3%/3years	63	NA	Hoarseness, ICHT, hypopharingeal extension, Gender	NO
Herrmann et al; 2016 (present study)	55	inferior border of the cricoid cartilage to 22 cm from incisors	RCT	Various: Cis/carboplatin,5-FU,taxotere (32)	28-72/1.2-2; HDR-Boost: 6/3 (2)	52%/3years	23	5	ICHT, RT dose ≥56Gy, Tumor grade	NO

Stuschke et al. [16] published in 1999 their data on 17 CEC patients treated with ICHT followed by concurrent CHT and high-dose RT for locally advanced SCC CEC. Three-year survival in their cohort was 24%. During ICHT, patients received either treatment with 5-FU (5FU/leucovorin/cisplatin/etoposide [FLEP protocol]) followed by cisplatin/etoposide during concurrent CRT, or alternatively 5-FU/leucovorin/cisplatin for ICHT and 5-FU/cisplatin during concurrent CRT. Hematological toxicities after FLEP were observed in 36% of patients. In a follow-up study of 55 patients by the same group [20], 3-year survival rate in patients treated with FLEP ICHT (*n* = 25) was 37% and in those treated with 5-FU/L/P ICHT (*n* = 22) 18%. Patients with ICHT other than these two protocols (*n* = 8) had a 3-year survival rate of 31%. Differences were statistical not significant (*p* = 0.42). Zhang et al. [29] recently published data of 102 patients with CEC, treated with definitive CRT. Within this cohort, 18 patients (17.6%) received platin- based ICHT. In contrast to our study, for patients that received ICHT, 3-year OS and PFS were significantly worse than in patients who did not receive ICHT (11.1 vs. 45.5%, *p* = 0.016; 11.1 vs. 40.5%, *p* = 0.019). Their explanation of why ICHT was conversely related to survival was, that 17 out of 18 patients in the ICHT group had stage III disease. In our study, for patients receiving ICHT, 3-year LRC was 41%, DFS 25% and OS 34%, which was superior to Zhang’s and similar to Stuschke’s outcome. Most frequent ICHT complications in our study included significantly higher-grade two hematological toxicities (*p* = 0.04) than the group with no ICHT. But ICHT had no impact on increasing dysphagia, odynophagia and skin reactions (*p* = 0.052). These results in CEC stand in contrast to the experience in locoregionally advanced head and neck squamous cell cancer (HNSCC). There ICHT remains a subject of intense debate in the management of HNSCC. No overall survival benefit was identified from the ICHT [30]. The large, meta-analysis of chemotherapy on head and neck cancer (MACH-NC) of individual patient analysis of 17,346 patients from 93 randomized trials conducted between 1965 and 2000 reported first in 2000 [31] and then updated in 2009 [11], by Pignon et al. ICHT significantly improved the rate of distant metastases (HR, 0.73; 95% CI, 0.61 to 0.88; *P* = 0.001) but did not influence locoregional failure.

With regards to the total radiation dose, there has been a tendency in CEC - in analogy to HNSCC - to use higher doses of radiation up to 66–70Gy. Retrospective studies [7, 12, 15, 16, 18, 19, 29] have shown that higher dose of radiation might be associated with improved outcome in CEC. Zhang et al.’s [29] study revealed that patients with stage II - III esophageal cancer treated with concurrent CRT with a radiation dose >51Gy (54–64.8Gy) had better LRC and OS than those treated with ≤51Gy. The median dose in the lower and higher dose groups was 30Gy (range, 30–51Gy) and 59.4Gy (range, 54–64.8Gy), respectively. Patients in the higher dose group had a statistically significant better 3-year LCR (36 vs.19%; *p* = 0.011), DFS (25 vs.10%; *p* = 0.004), and OS (13 vs. 3%; *p* = 0.054) rate. Wang et al. [18] reported that OS, cause specific survival (CSS), and local relapse-free survival (LRFS) rates were significantly higher in patients receiving a radiation dose >50Gy than in those < 50Gy. Total radiation dose was the only independent factor associated with improved local control and OS. Our data confirm that. Our study confirms these findings: multivariate analysis showed that radiation dose >56Gy was a significant positive predictive factor for DFS (*p* = 0.03) and OS (*p* = 0.006). However, Huang et al. [13] found no difference between high-dose RCT of 70Gy compared to RCT to 54Gy. They have treated CEC patients with two different treatment protocols with 54Gy in 20 fractions within 4 weeks, combined with 5-FU and either mitomycin or cisplatin vs. 70Gy in 35 fractions within 7 weeks to the primary tumor and elective nodes, with high-dose cisplatin.

Looking at other potential prognostic factors, no consistency exists within the literature. Our study revealed that tumor grade was a prognostic factor for OS (*p* = 0.03). T and N categories were not statistically significant prognostic factors for LRC, DFS or OS (*p* > 0.05). We assume, our cohort was too small to detect a statistical significant difference. In a study by Huang et al. [13], female gender and older age might predict for a better outcome, but a statistical significant difference could not be demonstrated. In Wang et al.’s [18] study, radiation dose (>50Gy vs. <50Gy) was the only factor associated with OS (*p =* 0.006), CSS (*p =* 0.003), and LRFS (*p =* 0.001) and tumor stage was the only factor associated with DFS (*p =* 0.007). In Zhang et al.’s [29] study, multivariate analysis revealed that gender and hoarseness were independent prognostic factors related to OS and PFS. Hoarseness (HR = 2.834; *p* = 0.002) was the only independent prognostic factor affecting LRFFS. Yamada et al.’s [15] study showed that performance status (*p* < 0.01) and tumor length (*p* < 0.01) were independent prognostic factors. The role of involvement of human papillomavirus (HPV) as a prognostic factor in the setting of SCC esophageal carcinoma remains unclearly defined. In oropharyngeal lesions, HPV-positivity has shown to be a strong positive prognostic factor in patient outcomes [32–35], whereas HPV in esophageal SCC does not appear to be a significant etiologic agent [36]. Furihata et al. [37] have shown that, HPV infection in SCC esophageal carcinoma was a poor prognostic indicator. In contrast, a recent series by Cao et al. [38], showed improved overall and disease-free survival in SCC esophageal carcinoma in patients with HPV- positive tumors. Several other studies still have failed to show any significant association between HPV infection and patient survival [39–41]. Since in the present cohort systematic testing of the HPV status for CEC patients has not been a standard procedure within the work up process during the period of investigation, no data was available for the HPV status.

As for other studies already published on this topic, some important limitations should be acknowledged in our study. It is of retrospective nature, and therefore, we could underestimate the toxicity data, which is an important considerable factor, when an intensification of the treatment is planned (with ICHT and/or dose escalation). Moreover, the multicenter data collection allowed to increase the number of patients, but it added also some bias related to local treatment standards. Nevertheless, we think that our study is of interest, as considerable practice variations exist worldwide in using definitive RT with or without CHT for the management of CEC. Our results add new aspects to the data already available in the literature. We think that it could be hypothesis generating for a prospective study, exploring the role of ICHT and/or dose escalation in the treatment of CEC.

## Conclusion

Results of our study confirm that definitive CRT with or without ICHT can be considered as an alternative to surgery in the treatment of CEC, as the 3-year outcomes are very encouraging and the toxicity acceptable. ICHT and cumulative RT doses > 56Gy were associated with a better outcome. Our study supports the design of prospective studies exploring schedules of treatment intensification including ICHT and RT doses > 56Gy in CEC patients.

## Abbrevations

(CEC): Cervical esophageal cancer
(CHT): Chemotherapy
(CI): Confidence interval
(CIS): Carcinoma in situ
(CRT): Chemo-radiation-therapy
(CSS): Cause specific survival
(CTCAE-NCI v.4.0): Common terminology criteria for adverse events, version 4.0
(DFS): Disease-free survival
(FLEP): 5FU/leucovorin/cisplatin/etoposide
(HDR-BT): High dose rate brachytherapy
(HNSCC): Head and neck squamous cell cancer
(HPV): Human papillomavirus
(HR): Hazard ratio
(ICHT): Induction chemotherapy
(IMRT): Intensity-modulated radiation therapy
(LRC): Loco-regional control
(LRFS): Local relapsefree survival
(MVA): Multivariable analysis
(NA): Not available
(OS): Overall survival
(PFS): Progression free survival
(RT): Radiotherapy
(SCC): Squamous-cell carcinoma
(UVA): Univariable analysis

## References

[1] Mendenhall WM, Sombeck MD, Parsons JT, Kasper ME, Stringer SP, Vogel SB (1994). Management of cervical esophageal carcinoma. Semin Radiat Oncol.

[2] Bosset JF, Gignoux M, Triboulet JP, Tiret E, Mantion G, Elias D, Lozach P, Ollier JC, Pavy JJ, Mercier M (1997). Sahmoud T.N chemoradiotherapy followed by surgery compared with surgery alone in squamous-cell cancer of the esophagus. Engl J Med.

[3] Urba SG, Orringer MB, Turrisi A, Iannettoni M, Forastiere A, Strawderman M (2001). Randomized trial of preoperative chemoradiation versus surgery alone in patients with locoregional esophageal carcinoma. J Clin Oncol.

[4] Kaklamanos IG, Walker GR, Ferry K, Franceschi D, Livingstone AS (2003). Neoadjuvant treatment for resectable cancer of the esophagus and the gastroesophageal junction: a meta-analysis of randomized clinical trials. Ann Surg Oncol.

[5] Allemann P, Mantziari S, Wagner D, Digklia A, Ozsahin E, De Bari B, Dorta G, Godat S, Montserrat F, Sempoux C, Brunel C, Demartines N, Schäfer M (2016). Curative treatment for esophageal cancer: results of a multidisciplinary consensus. Rev Med Suisse.

[6] Grass GD, Cooper SL, Armeson K, Garrett-Mayer E, Sharma A (2015). Cervical esophageal cancer: a population-based study. Head Neck.

[7] Tong DK, Law S, Kwong DL, Wei WI, Ng RW, Wong KH (2011). Current management of cervical esophageal cancer. World J Surg.

[8] Hoeben A, Polak J, Van De Voorde L, Hoebers F, Grabsch HI (2016). deVos-Geelen. Cervical esophageal cancer: a gap in cancer knowledge. J Ann Oncol.

[9] Minsky BD, Pajak TF, Ginsberg RJ, Pisansky TM, Martenson J, Komaki R, Okawara G, Rosenthal SA, Kelsen DP (2002). INT 0123 (radiation therapy oncology group 94-05) phase III trial of combined-modality therapy for esophageal cancer: high-dose versus standard-dose radiation therapy. J Clin Oncol.

[10] Cooper JS, Guo MD, Herskovic A, Macdonald JS, Martenson JA, Al-Sarraf M, Byhardt R, Russell AH, Beitler JJ, Spencer S, Asbell SO, Graham MV, Leichman LL (1999). Chemoradiotherapy of locally advanced esophageal cancer: long-term follow-up of a prospective randomized trial (RTOG 85-01). radiation therapy oncology group. JAMA.

[11] Pignon JP, le Maître A, Maillard E, Bourhis J, MACH-NC collaborative group (2009). Meta-analysis of chemotherapy in head and neck cancer (MACH-NC): an update on 93 randomised trials and 17,346 patients. Radiother Oncol.

[12] Burmeister BH, Dickie G, Smithers BM, Hodge R, Morton K (2000). Thirty-four patients with carcinoma of the cervical esophagus treated with chemoradiation therapy. Arch Otolaryngol Head Neck Surg.

[13] Huang SH, Lockwood G, Brierley J, Cummings B, Kim J, Wong R, Bayley A (2008). Ringash effect of concurrent high-dose cisplatin chemotherapy and conformal radiotherapy on cervical esophageal cancer survival. J Int J Radiat Oncol Biol Phys.

[14] Uno T, Isobe K, Kawakami H, Ueno N, Shimada H, Matsubara H, Okazumi S, Nabeya Y, Shiratori T, Kawata T, Ochiai T, Ito H (2007). Concurrent chemoradiation for patients with squamous cell carcinoma of the cervical esophagus. Dis Esophagus.

[15] Yamada K, Murakami M, Okamoto Y, Okuno Y, Nakajima T, Kusumi F, Takakuwa H, Matsusue S (2006). Treatment results of radiotherapy for carcinoma of the cervical esophagus. Acta Oncol.

[16] Stuschke M, Stahl M, Wilke H, Walz MK, Oldenburg AR, Stüben G, Jahnke K, Seeber S, Sack H (1999). Induction chemotherapy followed by concurrent chemotherapy and high-dose radiotherapy for locally advanced squamous cell carcinoma of the cervical oesophagus. Oncology.

[17] Mendenhall WM, Parsons JT, Vogel SB, Cassisi NJ, Million RR (1988). Carcinoma of the cervical esophagus treated with radiation therapy. Laryngoscope.

[18] Wang S, Liao Z, Chen Y, Chang JY, Jeter M, Guerrero T, Ajani J, Phan A, Swisher S, Allen P, Cox JD, Komaki R (2006). Esophageal cancer located at the neck and upper thorax treated with concurrent chemoradiation: a single-institution experience. J Thorac Oncol.

[19] Cao CN, Luo JW, Gao L, Xu GZ, Yi JL, Huang XD, Wang K, Zhang SP, Qu Y, Li SY, Xiao JP, Zhang Z (2016). Intensity-modulated radiotherapy for cervical esophageal squamous cell carcinoma: clinical outcomes and patterns of failure. Eur Arch Otorhinolaryngol.

[20] Gkika E, Gauler T, Eberhardt W, Stahl M, Stuschke M, Pöttgen C (2014). Long-term results of definitive radiochemotherapy in locally advanced cancers of the cervical esophagus. Dis Esophagus.

[21] Gebski V, Burmeister B, Smithers BM, Foo K, Zalcberg J, Simes J, Australasian gastro-intestinal trials group (2007). Survival benefits from neoadjuvant chemoradiotherapy or chemotherapy in oesophageal carcinoma: a meta-analysis. Lancet Oncol.

[22] Tepper J, Krasna MJ, Niedzwiecki D, Hollis D, Reed CE, Goldberg R, Kiel K, Willett C, Sugarbaker D, Mayer R (2008). Phase III trial of trimodality therapy with cisplatin, fluorouracil, radiotherapy, and surgery compared with surgery alone for esophageal cancer: CALGB 9781. J Clin Oncol.

[23] van Hagen P, Hulshof MC, van Lanschot JJ, Steyerberg EW, van Berge Henegouwen MI, Wijnhoven BP, Richel DJ, Nieuwenhuijzen GA, Hospers GA, Bonenkamp JJ, Cuesta MA, Blaisse RJ, Busch OR, ten Kate FJ, Creemers GJ, Punt CJ, Plukker JT, Verheul HM, Spillenaar Bilgen EJ, van Dekken H, van der Sangen MJ, Rozema T, Biermann K, Beukema JC, Piet AH, van Rij CM, Reinders JG, Tilanus HW, van der Gaast A, CROSS Group (2012). Preoperative chemoradiotherapy for esophageal or junctional cancer. N Engl J Med.

[24] Wu AJ, Bosch WR, Chang DT, Hong TS, Jabbour SK, Kleinberg LR, Mamon HJ, Thomas CR, Goodman KA (2015). Expert consensus contouring guidelines for intensity modulated radiation therapy in esophageal and gastroesophageal junction cancer. Int J Radiat Oncol Biol Phys.

[25] Common Terminology Criteria for Adverse Events (CTCAE) Version 4.0. Available at: http://evs.nci.nih.gov/ftp1/CTCAE/CTCAE_4.03_2010-06-14_QuickReference_5x7.pdf; May 28, 2009

[26] Cao CN, Luo JW, Gao L, Xu GZ, Yi JL, Huang XD, Li SY, Xiao JP, Liu SY, Xu ZG, Tang PZ (2014). Primary radiotherapy compared with primary surgery in cervical esophageal cancer. JAMA Otolaryngol Head Neck Surg.

[27] Cao C, Luo J, Gao L, Xu G, Yi J, Huang X, Wang K, Zhang S, Qu Y, Li S, Xiao J, Zhang Z (2015). Definitive intensity-modulated radiotherapy compared with definitive conventional radiotherapy in cervical oesophageal squamous cell carcinoma. Radiol Med.

[28] Cao C, Luo J, Gao L, Xu G, Yi J, Huang X, Wang K, Zhang S, Qu Y, Li S, Xiao J, Zhang Z (2015). Definitive radiotherapy for cervical esophageal cancer. Head Neck.

[29] Zhang P, Xi M, Zhao L, Qiu B, Liu H, Hu YH, Liu MZ (2015). Clinical efficacy and failure pattern in patients with cervical esophageal cancer treated with definitive chemoradiotherapy. Radiother Oncol.

[30] Argiris A, Karamouzis MV, Raben D, Ferris RL (2008). Head and neck cancer. Lancet.

[31] Pignon JP, Bourhis J, Domenge C, Designé L (2000). Chemotherapy added to locoregional treatment for head and neck squamous-cell carcinoma: three meta-analyses of updated individual data. MACH-NC collaborative group. Meta-analysis of chemotherapy on head and neck cancer. Lancet.

[32] Ang KK, Harris J, Wheeler R, Weber R, Rosenthal DI, Nguyen-Tân PF, Westra WH, Chung CH, Jordan RC, Lu C, Kim H, Axelrod R, Silverman CC, Redmond KP, Gillison ML (2010). Human papillomavirus and survival of patients with oropharyngeal cancer. N Engl J Med.

[33] Weinberger PM, Yu Z, Haffty BG, Kowalski D, Harigopal M, Brandsma J, Sasaki C, Joe J, Camp RL, Rimm DL, Psyrri A (2006). Molecular classification identifies a subset of human papillomavirus--associated oropharyngeal cancers with favorable prognosis. J Clin Oncol.

[34] Gillison ML, Koch WM, Capone RB, Spafford M, Westra WH, Wu L, Zahurak ML, Daniel RW, Viglione M, Symer DE, Shah KV, Sidransky D (2000). Evidence for a causal association between human papillomavirus and a subset of head and neck cancers. J Natl Cancer Inst.

[35] Fakhry C, Westra WH, Li S, Cmelak A, Ridge JA, Pinto H, Forastiere A, Gillison ML (2008). Improved survival of patients with human papillomavirus-positive head and neck squamous cell carcinoma in a prospective clinical trial. J Natl Cancer Inst.

[36] Ludmir EB, Stephens SJ, Manisha Palta M, Willett CG, Czito BG (2015). Human papillomavirus tumor infection in esophageal squamous cell carcinoma. J Gastrointest Oncol.

[37] Furihata M, Ohtsuki Y, Ogoshi S, Takahashi A, Tamiya T, Ogata T (1993). Prognostic significance of human papillomavirus genomes (type-16, -18) and aberrant expression of p53 protein in human esophageal cancer. Int J Cancer.

[38] Cao F, Han H, Zhang F, Wang B, Ma W, Wang Y, Sun G, Shi M, Ren Y, Cheng Y (2014). HPV infection in esophageal squamous cell carcinoma and its relationship to the prognosis of patients in northern China. Scientific World Journal.

[39] Dreilich M, Bergqvist M, Moberg M, Brattström D, Gustavsson I, Bergström S, Wanders A, Hesselius P, Wagenius G, Gyllensten U (2006). High-risk human papilloma virus (HPV) and survival in patients with esophageal carcinoma: a pilot study. BMC Cancer.

[40] Antonsson A, Nancarrow DJ, Brown IS, Green AC, Drew PA, Watson DI, Hayward NK, Whiteman DC, Australian Cancer Study (2010). High-risk human papillomavirus in esophageal squamous cell carcinoma. Cancer Epidemiol Biomarkers Prev.

[41] Hippeläinen M, Eskelinen M, Lipponen P, Chang F, Syrjänen K (1993). Mitotic activity index, volume corrected mitotic index and human papilloma-virus suggestive morphology are not prognostic factors in carcinoma of the oesophagus. Anticancer Res.

[42] Chou SH, Li HP, Lee JY, Huang MF, Lee CH, Lee KW (2010). Radical resection or chemoradiotherapy for cervical esophageal cancer?. World J Surg.

[43] Ma JB, Song YP, Yu JM, Zhou W, Cheng EC, Zhang XQ, Kong L (2011). Feasibility of involved-field conformal radiotherapy for cervical and upper-thoracic esophageal cancer. Onkologie.

[44] Tu L, Sun L, Xu Y, Wang Y, Zhou L, Liu Y, Zhu J, Peng F, Wei Y, Gong Y (2013). Paclitaxel and cisplatin combined with intensity-modulated radiotherapy for upper esophageal carcinoma. Radiat Oncol.

[45] Ludmir EB, Palta M, Zhang X, Wu Y, Willett CG, Czito BG (2014). Incidence and prognostic impact of high-risk HPV tumor infection in cervical esophageal carcinoma. J Gastrointest Oncol.

